# EHMTI-0028. Occipital neuralgiform pain as multiple sclerosis relapse

**DOI:** 10.1186/1129-2377-15-S1-C49

**Published:** 2014-09-18

**Authors:** M Ozerden, A Soysal, N Kale, E Coban

**Affiliations:** 1 Department of 3rd Neurology, Bakirkoy Research and Training Hospital for Neurology, Psychiatry and Neurosurgery, Istanbul, Turkey

## 

Occipital neuralgia(ON) is a rare presentation and might be due to multiple causes including occipital nerve pathologies and limit daily function of patients and have a severe impact on their quality of life (QoL). The clinical cahrecteristics of ON include paroximal painful attacks of electric shock- like sensation, occuring spontaneously or evoked by stimuli in specific tigger areas. Cervical cord demyelination might rareley cause ON. In this case report we would like to report a clinically definite relapsing-remitting multiple sclerosis (MS) patient who experienced occipital neuralgiform pain with a contrast enhancing demyelinating lesion on C2 cervical cord. We would like to discuss a rare case of occipital neuralgia presenting as an MS relapse. Although patients with multiple sclerosis have an increased incidence of headaches, new onset headaches might be related to a new active lesion and patient sholud be evaluated considering this possibility.

No conflict of interest.

